# Posterior Communicating Artery Aneurysm With Referred Tooth Pain: A Case Report

**DOI:** 10.7759/cureus.46027

**Published:** 2023-09-26

**Authors:** Thomas J Jacob, Shivankshi Berry, Sindhu H Peketi, Adriel Abraham, Jai Joshi, Shoaib Rashid, Leonid Rankov

**Affiliations:** 1 Internal Medicine, New York Institute of Technology College of Osteopathic Medicine, Old Westbury, USA; 2 Medicine, Dayanand Medical College and Hospital, Ludhiana, IND; 3 Internal Medicine, Nassau University Medical Center, East Meadow, USA; 4 Surgery, New York Institute of Technology College of Osteopathic Medicine, Old Westbury, USA; 5 Radiology, Nassau University Medical Center, East Meadow, USA

**Keywords:** diplopia, oculomotor nerve (cn iii) palsy, referred pain, aneurysm, posterior communicating artery (pcom)

## Abstract

Oculomotor nerve (CN III) palsy (ONP) has multiple etiologies, with aneurysms and ischemic injury being the two leading causes. The presentations of these conditions differ, as aneurysms commonly manifest with pupillary involvement, while ischemic-related ONP often leads to a pupil-sparing presentation. We present a 63-year-old African American male with a history of sickle cell trait, ocular sickle cell disease, and untreated hypertension that develops "down and out" left eye with a mid-dilated pupil unresponsive to light. However, the patient developed severe left upper tooth pain after the onset of the eye pain, which progressed to ONP. The patient's dental and radiographic evaluation did not indicate any obvious source for his tooth pain. Magnetic resonance imaging (MRI) and magnetic resonance angiography (MRA) of the head revealed a 7-mm saccular aneurysm with a 2-mm neck arising from the left posterior communicating artery (PCOM) aneurysm, and neurovascular surgical intervention was initiated. This case highlights the potential of referred tooth pain as an early symptom in patients with PCOM aneurysm, which physicians should be vigilant about and consider as a potential indicator of the condition. Therefore, collaboration between different specialties, including ophthalmology, neurology, neurosurgery, and dental care, is necessary to formulate a comprehensive treatment plan that effectively addresses the patient's specific needs and challenges.

## Introduction

Oculomotor nerve (CN III) palsy (ONP) has a variety of etiologies, including traumatic, autoimmune, infectious, ischemic destruction, or compressive masses such as tumors and aneurysms [1]. CN III dysfunction can present with varying symptoms, such as the classic "down and out" resting position, diplopia, mono-ocular blurry vision, eye pain, headache, or a dilated pupil [2]. Ptosis results from the involvement of the levator palpebrae superioris muscle, while diplopia in CN III dysfunction is due to the involvement of the superior rectus, inferior rectus, medial rectus, and inferior oblique [3]. The fixed dilated pupil appearance results from affecting the sphincter papillae muscle [3]. Posterior communicating artery (PCOM) aneurysms represent 50% of all internal carotid artery aneurysms. However, not all present with a typical subarachnoid hemorrhage, but some may present with ONP or non-traumatic subdural hemorrhage. Third cranial nerve palsy is the most common cranial nerve injury caused by PCOM aneurysm, and the eye pain associated with CN III compression is typically retro-orbital. However, we describe a patient with a PCOM artery aneurysm with ONP and referred tooth pain.

## Case presentation

A 63-year-old African American male with a medical history of sickle cell trait, sickle cell retinopathy, and untreated hypertension was referred from urgent care to evaluate left eye as well as tooth pain and tenderness with associated blurry vision for the past five days. The patient had been experiencing decreasing visual acuity in both eyes for the past year. However, in the past five days, he reported severe left upper tooth pain associated with pain over his eye, which made him seek medical care. He also endorsed double vision and increased left eye lacrimation.

On a physical examination at admission to our hospital, the patient had an elevated blood pressure of 170/90 mmHg. Cranial nerve examination was significant for left eye ptosis, large unreactive left-sided pupil, and down and outward gaze palsy of the left eye. The oral exam was within normal limits; however, the patient had tenderness over teeth #13 and tenderness on the face in the corresponding region extending to the eye. However, no caries or inflammation was found, and no triggers were noticed.

The initial CT of the head showed no acute intracranial hemorrhage or extra-axial collections. CT of the orbit, ear, and fossa showed no evidence of orbital inflammation. A carotid ultrasound showed no significant stenosis on either side by velocity criteria.

After his blood pressure was adequately controlled and with a suboptimal response to pain, we consulted a dental surgeon. On further dental examination, tooth #13 did not have evidence of caries but was tender and non-erythematous/edematous. The radiographic evaluation indicated no caries or periapical radiolucency (PARL), intact periodontal ligament (PDL), and bone loss on teeth #14. An occlusal adjustment was performed on tooth #13, and the patient reported only mild improvement in pain.

**Figure 1:**
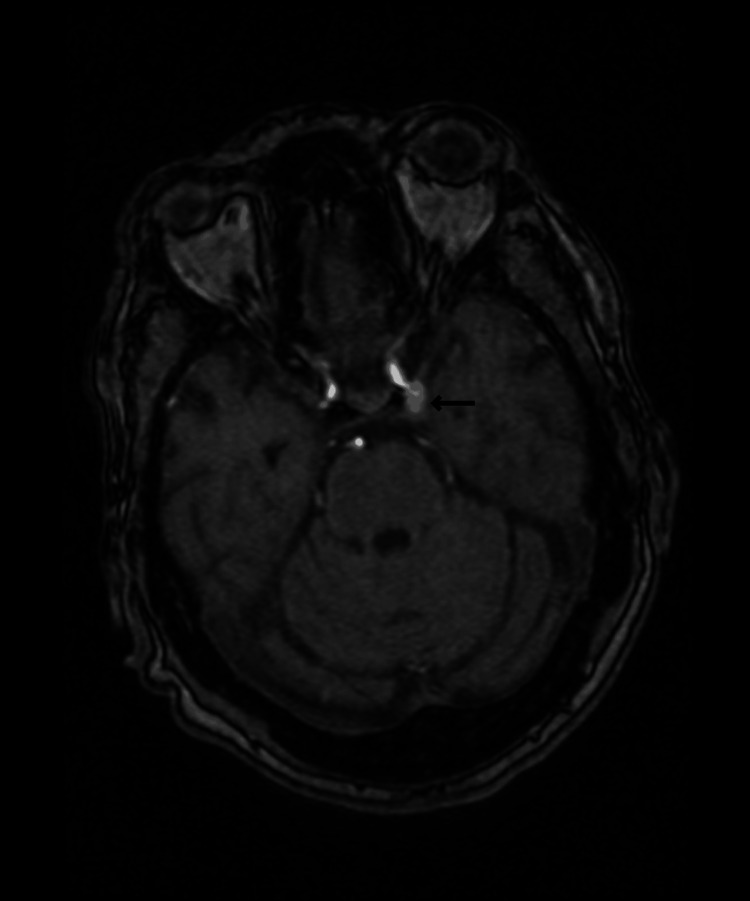
MRA of the head. There is an outpouching arising from the posterior aspect of the terminal segment of the left internal carotid artery compatible with a PCOM aneurysm that measures 7 mm in anteroposterior (AP) dimension, with a narrow neck measuring 2 mm MRA: Magnetic resonance angiogram; PCOM: Posterior communicating artery.

Magnetic resonance imaging (MRI) and magnetic resonance angiography (MRA) of the brain were performed to determine the compressive cause of CN III due to pupillary involvement and pain associated with the V2 region. A 7-mm saccular aneurysm with a 2-mm neck arising from the left PCOM was found on the MRA of the head, which can be visualized in Figure 1. The patient was transferred to a different hospital for balloon-assisted coiling. Post-procedurally, the patient endorsed improved eye pain, tooth pain, and diplopia.

## Discussion

The oculomotor nerve has two divisions: the superior division, which supplies the levator palpebrae and the superior rectus muscles, and the inferior division, which supplies the medial and inferior rectus muscles, the inferior oblique muscle, and the pupillary sphincter [4]. Damage to these divisions can lead to the unopposed action of the lateral rectus and superior oblique muscles, resulting in the fixed eye in the down and out position. Diplopia can occur due to ocular deviations, causing the image projection to fall on an extrafoveal point [5]. Furthermore, the impairment of the levator palpebrae superioris muscle and the parasympathetic innervation to the pupillary sphincter results in ptosis and mydriasis, respectively [6]. In this case, we have to explore the two significant etiologies of ONP: aneurysms and ischemia-induced.

Aneurysm-related ONP and ischemic/sickle cell-related ONP are different etiologies that can result in similar clinical presentations. In aneurysm-related ONP, the involvement of CN III occurs due to direct compression by an aneurysm, arterial pulse transmission, or nerve edema from venous obstruction [7]. The parasympathetic fibers run along the dorsomedial surface of the oculomotor nerve, so they can be easily compressed by a PCOM aneurysm, causing oculomotor dysfunctions, which may cause ptosis, diplopia, and pupillary dilation [8]. On the other hand, ischemic/sickle cell-related CN III palsy refers to the impairment of CN III due to ischemic events or microvascular disease that leads to a pupil-sparing ONP [9]. Pupil-sparing ONP refers to the involvement of the oculomotor nerve (CN III) without affecting the pupillary function because the core of the oculomotor nerve is damaged in ischemic diseases [10]. However, pupil-sparing ONP may arise as an early symptom of an enlarging posterior communicating artery aneurysm that can rupture [8]. In this case, the presence of old sickle cell retinopathy and the subsequent discovery of a saccular aneurysm arising from the left PCOM indicate the coexistence of both etiologies, highlighting the complexity of the patient's condition.

Referred tooth pain refers to pain perceived in a tooth that originates from a source outside the tooth itself. In this case, the patient experienced left upper tooth pain more significant than diplopia, which made him seek medical attention. The dental evaluation revealed tenderness on the percussion of tooth #13. However, no evidence of dental caries was found, suggesting that the tooth may not be the source of the pain. One proposed mechanism of referred pain is the theory of convergence, where a single second-order neuron receives nociceptive input from various primary somatic or visceral afferents originating from different locations (e.g., the eye or sinuses) [11]. However, the brain cannot precisely determine the exact source of the stimulus, resulting in a misinterpretation and incorrect localization of the sensation [11]. The precise mechanism of the referred tooth pain remains unclear in this case; however, it is presumed to be due to compression of the V2 portion of the trigeminal nerve by PCOM, causing pain in the region of tooth #13 and on the corresponding areas of the face. Considering and evaluating potential sources of referred pain is crucial when diagnosing and managing dental symptoms. This case suggests that referred tooth pain could be one of the atypical symptoms that patients might experience due to a PCOM artery aneurysm, especially in patients with poor pain localization.

Given the presence of a saccular aneurysm and its potential association with the patient's symptoms, neurovascular surgery intervention would be crucial to address the aneurysm and relieve compression on CN III. Also, managing the patient's underlying medical conditions, such as optimizing blood pressure, is paramount to mitigating further damage. In cases of sickle cell disease, addressing the underlying disease and providing supportive care, such as hydration and pain management, are necessary. The management of an unruptured aneurysm involves three approaches: conservative treatment, surgical clipping, and endovascular techniques [12]. The management choice is determined by factors such as the size of the aneurysm and the potential complications associated with each treatment method [12]. Surgical treatment is preferable if the aneurysm is large, especially when it is causing a mass effect on the oculomotor nerve, aneurysms with unfavorable fundus, and those associated with fetal PCOM artery origin. Therefore, collaboration between different specialties, including ophthalmology, neurology, neurosurgery, and dental care, is crucial in developing a comprehensive treatment plan that addresses the specific needs and challenges of the patient.

## Conclusions

Oculomotor nerve palsy and referred tooth pain are two distinct clinical presentations that can arise from different etiologies. In the case presented, a patient with a PCOM artery aneurysm experienced both ONP and referred tooth pain. The mechanical compression from the aneurysm most likely led to the ONP. Referred tooth pain, in this case, is also potentially due to the compressive effect of the aneurysm on the V2 portion of the trigeminal nerve. The management of this patient required a multidisciplinary approach involving ophthalmology, neurology, neurosurgery, and dental care. Accurate diagnosis and appropriate intervention were crucial in addressing the aneurysm and relieving compression on CN III.
